# Knowledge, Attitudes, and Perceptions of Jordanians Toward Adopting and Using Telemedicine: National Cross-sectional Study

**DOI:** 10.2196/41499

**Published:** 2022-11-04

**Authors:** Rand Murshidi, Muhammad Hammouri, Hana Taha, Razi Kitaneh, Mahmoud Alshneikat, Abdallah Al-Qawasmeh, Ahmad Al-Oleimat, Leen Al-Huneidy, Yazan Al-Huneidy, Abdallah Al-Ani

**Affiliations:** 1 Department of Dermatology School of Medicine The University of Jordan Amman Jordan; 2 School of Medicine The University of Jordan Amman Jordan; 3 Department of Basic Medical Sciences The Hashemite University Zarqa Jordan; 4 Department of Internal Medicine King Hussein Medical Center Amman Jordan; 5 Office of Scientific Affairs and Research King Hussein Cancer Center Amman Jordan

**Keywords:** Jordan, telemedicine, KAP study, COVID-19, telehealth, regression model, user perception, online survey, privacy, health care system

## Abstract

**Background:**

Due to the upsurge of COVID-19, nations are increasingly adopting telemedicine programs in anticipation of similar crises. Similar to all nations worldwide, Jordan is implementing efforts to adopt such technologies, yet it is far from complete.

**Objective:**

This study aims to assess the knowledge, attitudes, and perceptions of Jordanians toward telemedicine, to identify key factors predisposing individuals to its use or acting as barriers to its implementation.

**Methods:**

We implemented a cross-sectional design using an online, self-administered questionnaire executed in Google Forms and distributed through social media. Differences in knowledge and attitude scores were examined using independent sample *t* tests and ANOVA. A multivariate linear regression model was computed to assess predictors of awareness toward telemedicine.

**Results:**

A total of 1201 participants fully completed the questionnaire. Participants were characterized by a mean age of 36.3 (SD 14.4) years and a male-to-female ratio of nearly 1:1. About 50% (619/1201, 51.5%) of our studied population were aware of telemedicine, while nearly 25% (299/1201, 24.9%) declared they had observed it in action. Approximatively 68% (814/1201, 67.8%) of respondents were willing to use telemedicine. The majority of the sample portrayed favorable and positive views toward telemedicine. Higher educational degrees, living in urban districts, and having a higher perception of electronic usage ability were associated with higher knowledge and better attitudes toward telemedicine (all *P*<.05). The multivariate linear regression analysis demonstrated that perceived ability to use electronics was associated with positive attitudes (*β*=0.394; 95% CI 0.224 to 0.563), while living in Southern Jordan predicted poor attitudes toward telemedicine (*β*=–2.896; 95% CI –4.873 to –0.919).

**Conclusions:**

Jordanians portray favorable perceptions of telemedicine. Nonetheless, concerns with regards to privacy, medical errors, and capacity for accurate diagnoses are prevalent. Furthermore, Jordanians believe that integrating telemedicine within the health care system is not applicable due to limited resources.

## Introduction

Telemedicine is defined by the World Health Organization as the use of information and communication technologies to promote health, provide medical care, exchange medical information, and educate health care providers and patients over long distances [1]. It was properly integrated into clinical practice as early as 1959 within the field of psychiatry [2]. In recent years, there has been expeditious adoption and rising interest by policy makers of this technology across most disciplines of medical care worldwide [3-5]. Those changes were a direct cause of and paralleled by the COVID-19 pandemic, as the need for ingenious ways to effectively provide health care while maintaining implementations of social distancing and policies to limit the spread of the virus was imperative [6].

Although the literature recognizes the myriad of benefits of telemedicine, such as reducing travel time, decreasing consultation fees, and increasing access to medical services to residents of remote, low-resource regions [7-9], impediments to the proper implementation of this technology worldwide still exist, especially within low-resource settings. Namely, hurdles related to cost, lack of technical staff, privacy of information, and resistance to change were among the most commonly reported [10-12].

Along those challenges, there are some aspects that are particularly important in the Arab context, specifically those pertaining to social and religious restrictions [13,14]. One example of this is the case of dealing with the opposite sex (ie, when a male health care provider has to cooperate with a female clinical staff or patient and vice versa). Although permitted in Islam under specific rules, the traditional beliefs of parties involved may prohibit the use of telemedicine services. Such a phenomenon is considered one of the top challenges of implanting telemedicine in Saudi health care [15].

In the Arab world, the concept of telemedicine is still relatively new, and applications of this technology are still limited [16,17]. Jordan as a country is no different to this situation as early attempts to carry out telemedicine projects of limited reach date back to 2008 [18]. Other small-scale projects, including digital mental health services and counseling of pediatric asthma patients, also took place before and in response to the COVID-19 pandemic [19-22].

To date, some of the well-established telemedicine programs in Jordan include the Hakeem Portal, which provides patients with the service of booking outpatient follow-up consultations within public health institutions [23]. Other programs include the King Hussein Cancer Center programs for retinoblastoma diagnosis and management as well as consultation services for general cancer cases [24,25], specialized telehealth consultations in some of the private sector hospitals [26], and finally, independent service providers like that of “AlTibbi,” a mobile health service [27]. Nonetheless, successful nationwide adoption of telemedicine is still far from complete. Taking into consideration the reciprocal nature of this service that requires good technological literacy, supporting infrastructure, an adequate level of patient education, and resistance to change in both staff and patients [28], successful implementation requires a thorough understanding of its tenants and evaluation of the skills required from parties involved, specifically, the recipients and providers, and an assessment of the possibility of incorporating it into the existing system, all of which has not been addressed in the Jordanian population.

Hence, the primary aim of this study was to investigate the knowledge, attitudes, and perceptions of the Jordanian public toward telemedicine. The secondary aims of this study were to identify key predictive factors of telemedicine usage and the potential obstacles for implementation.

## Methods

### Study Setting, Design, and Sampling

This cross-sectional study was conducted in The Hashemite Kingdom of Jordan, an upper-middle-income country located in the Middle East. Jordan has a population of 10.3 million and a median age of 23.8 years as of 2022. We designed and distributed an online, self-administered questionnaire executed in Google Forms and distributed through social media. We adopted a convenience sampling technique in order to approach the target group of the study, which aimed to represent the adult general population (≥18 years old) across all genders, governorates, and nationalities of those living in Jordan. The questionnaire was distributed among the target population during January 2022 through multiple popular social media platforms (eg, Facebook, Twitter, and WhatsApp). Participants were encouraged to share the questionnaire among their friends and relatives for maximum reach and generalizability of results. Simultaneously, a printed self-administered version of the questionnaire was utilized and distributed in places where different groups of the target population, especially those who are less likely to be active on the aforementioned social media platforms, could be found, thus reducing biases introduced by online surveys. These locations included the Jordan University Hospital, King Abdullah University Hospital, Al-Bashir public hospital, and the Royal Medical Services, as these locations provide medical care to the greater majority of individuals in Jordan. Moreover, in-person data collection was initiated in public spaces including malls, shopping districts, and entertainment districts. It should be noted that the Jordan University Hospital is the largest academic center in Jordan and is the largest referral center for all of central Jordan serving over 4 million patients. Participants included in the study were those who gave informed consent, completed the whole questionnaire, and were ≥18 years of age.

### Questionnaire Development

The questionnaire was constructed after conducting an extensive literature review [29-33]. The questionnaire’s first domain inquired about relevant sociodemographic variables of potential participants. These factors included age, biological sex, educational level, governorate or residence, place of residence (rural/urban), marital status, number of children, type of insurance, job type (office, field, student, and retired), type of internet connection at home, presence of chronic illness, presence of regularly administered medications, and the type of hospital the patient attends for regular follow up (governmental, private, university hospital, military, or others, if applicable). Also, the first domain examined the perceived level of competency with handling electronics using a 10-point scale. The second and third domains were primarily influenced by an exploration of attitudes toward telemedicine in Saudi Arabia [30]. The second domain was concerned with assessing the participants’ level of knowledge regarding telemedicine and its services. It consisted of 8 dichotomous items with a score ranging from 0 to 8. Using the Kuder-Richardson formula, the alpha value for the knowledge domain was 0.677. The third and final domain consisted of 14 items that were used to assess the attitudes (ie, general perceptions, perceived benefits, and concerns) of the study participants toward the use of telemedicine and its applications. Each item was associated with a 4-point Likert scale (1 = strongly disagree, 2 = disagree, 3 = agree, 4 = strongly agree). A neutral option was removed as means to avoid neutral bias when using the Likert scale and ease the process of statistical analysis. The first 11 items of the third domain contributed to the attitudes score, ranging from 11 to 44. Similarly, the final 3 items of the third domain constituted the perceptions score, ranging from 3 to 12.

During pilot testing, the Cronbach alpha for the attitudes and perceptions scores were 0.82 and 0.74, respectively, thus ensuring proper internal consistency. The questionnaire’s content validity was ensured by a panel of experts in global and public health. Meanwhile, face validity was ensured through respondents’ feedback during pilot testing. Construct validity was examined using factor analysis. The Barret test for sphericity was significant at *P*<.001, and the Kaiser Meyer Olkin measure was 0.886. Using principal component analysis with direct oblimin rotation, a total of 6 factors had eigenvalues greater than 1 and explained 58.2% of the variance. Scree plot inspection demonstrated the point of inflexion after 3 components explained 42.7% of the variance. The other components were excluded as they managed to explain less than 5% of the variance. The 3 components corresponded to the knowledge, attitude, and perception domains.

The questionnaire was translated to Arabic to ensure comprehensibility, and it was translated back to English, all through the help of an expert translator. The final questionnaire included a total of 36 items (including demographics), with an approximate time of completion of 4 minutes.

### Statistical Analysis

#### Statistical Methods

Statistical analysis was conducted using SPSS version 24 (IBM Corp, Armonk, NY). Data were reported as frequencies (n) and percentages (%) or means (SDs) wherever applicable. Normality of data was tested using the Shapiro-Wilk and Kolmogorov Smirnov tests. Mean differences were examined using the independent sample *t* test or ANOVA. A multivariate linear regression model was computed to assess the predictors of attitudes score. All statistical tests were conducted with 95% CIs and a 5% error margin. A *P* value <.05 was considered statistically significant.

#### Power and Sample Size

Considering that there are no comparable studies from Jordan that can be used to calculate the appropriate sample size, the estimated sample size was calculated using GPower 3.1 and EpiInfo. At a power of 95%, α margin of error of 5%, and effect size of 30%, a sample of 580 participants was needed to demonstrate statistical differences of appropriate power.

### Ethical Considerations

This study was approved by the University of Jordan Scientific Committee.

## Results

### Demographics

A total of 1201 participants fully completed the questionnaire. Participants were characterized by a mean age of 36.3 (SD 14.4) years and a male-to-female ratio of nearly 1:1. Most participants had at least a university degree (ie, bachelor’s; 758/1201, 63.1%), lived in the capital of Jordan (743/1201, 61.9%), and resided within urban cities (1056/1201, 87.9%). Nearly 58% (690/1201, 57.5%) of all participants were married, of which 70.7% (488/1201) had at least 3 children. Furthermore, 28.6% (344/1201) held office-based occupations, 26.2% (315/1201) were unemployed, 26.0% (312/1201) were students, 19.2% (230/1201) held field occupations, 4.4% (53, 1201) were housewives, and 3.9% (47/1201) were retired.

In terms of electronic competency, 54.3% (652/1201) perceived that they were highly competent in dealing with computers or electronic tablets. The most common internet connection types reported were 4G standard routers (386/1201, 30.6%), followed by fiber optic internet (522/1201, 23.5%). The sociodemographic characteristics of the cohort are described in Table 1.

**Table 1:** Demographics of the recruited participants (n=1201).

Variable			Results, n (%)
**Gender**			
	Male	615 (51.2)	
	Female	586 (48.8)	
**Age (years)**			
	18-30	515 (42.9)	
	31-40	232 (19.3)	
	41-50	227 (18.9)	
	51-60	165 (13.7)	
	61-85	62 (5.2)	
**Educational level**			
	Primary	29 (2.4)	
	High school	209 (17.4)	
	University	758 (63.1)	
	Postgraduate (ie, master’s)	205 (17.1)	
**Area**			
	Capital (Amman)	743 (61.9)	
	Central	210 (17.5)	
	North	198 (16.5)	
	South	50 (4.2)	
**Residence**			
	Urban	1056 (87.9)	
	Rural	145 (12.1)	
**Relationship status**			
	Married	690 (57.5)	
	Single	511 (42.5)	
**Number of children**			
	0	57 (8.3)	
	1	51 (7.4)	
	2	94 (13.6)	
	≥3	488 (70.7)	
**Nature of work**			
	Office	344 (28.6)	
	Field	230 (19.2)	
	Retired	47 (3.9)	
	Student	312 (26.0)	
	Housewife	53 (4.4)	
	Unemployed	215 (17.9)	
**Type of internet connection**			
	Fiber	522 (23.5)	
	4G router	368 (30.6)	
	ADSL	45 (3.7)	
	Phone packets	184 (15.3)	
	Router and phone packets	81 (6.7)	
	None	1 (0.1)	
Comorbidities (yes)			280 (23.3)
Medications (yes)			359 (29.9)
**Perceived ability**			
	High (8-10)	652 (54.3)	
	Low (0-7)	549 (45.7)	

### Knowledge of Telemedicine

Table 2 shows that 51.5% (619/1201) had heard of the concept of telemedicine, while only 24.9% (299/1201) had observed it in action. Also, only a mere 14.2% (170/1201) had tried using telemedicine within their lifetime. Nonetheless, participants demonstrated favorable views regarding telemedicine, as the greater majority agreed that telemedicine reduces the number of medical staff (755/1201, 62.9%), reduces transportation costs and time (1127/1201, 93.8%), facilitates better health care for older adults (948/1201, 78.9%), allows you to remotely conduct follow-up health care logistics (eg, acquiring a prescription refill; 1018/1201, 84.8%), and enables remote and close follow-up of patients’ illnesses (824/1201, 68.6%).

**Table 2:** Participants’ responses on the telemedicine knowledge items.

Knowledge items	Agree, n (%)	Disagree, n (%)
Have you heard of telemedicine before?	619 (51.5)	582 (48.5)
Have you observed a telemedicine process previously?	299 (24.9)	902 (75.1)
Have you ever used telemedicine before?	170 (14.2)	1031 (85.8)
Telemedicine reduces the number of needed medical staff.	755 (62.9)	446 (37.1)
Telemedicine reduces transportation costs and time.	1127 (93.8)	74 (6.2)
Telemedicine facilitates care for older adult patients.	948 (78.9)	253 (21.1)
With telemedicine, you can remotely acquire a prescription, refill, or admission orders	1018 (84.8)	183 (15.2)
With telemedicine, you can closely follow up with your ongoing/chronic illnesses remotely.	824 (68.6)	377 (31.4)

### Attitudes Toward Telemedicine

With regards to participants’ attitudes, our cohort displayed mostly favorable attitudes toward telemedicine. The majority of participants believed that telemedicine is useful during a pandemic (1001/1201, 83.3%), is able to decrease the number of outpatient visits (1033/1201, 86.0%), can increase the speed of performing health care services (893/1201, 74.4%), mitigates health care costs (897/1201, 74.7%), and is able to provide specialized health care to underserved areas (841/1201, 70.0%). Overall, 67.8% (814/1201) of all participants were willing to use telemedicine for diagnosis or follow up. Table 3 displays participants’ attitudes and perceptions.

On the other hand, a significant number of participants believed that telemedicine cannot provide accurate diagnoses (810/1201, 67.4%) or comprehensive health care (757/1201, 63.0%). Moreover, the greater majority of participants believed that telemedicine is unable to reduce medical errors (889/1201, 74.0%). Most importantly, 43.5% (522/1201) of all participants believed that telemedicine may pose a threat to their information privacy. In terms of perceptions, most participants believed that Jordan does not have the capacity to implement telemedicine (626/1201, 60.4%); however, more than one-half (654/1201, 54.5%) of the cohort perceived telemedicine as the future of health care.

**Table 3:** Attitudes and perceptions of respondents toward telemedicine.

Attitudes and perceptions			Response categories, n (%)									Score, mean (SD)
			Strongly disagree		Disagree		Agree		Strongly agree			
**Attitudes**												
	Is telemedicine useful during a pandemic (eg, COVID-19)?	71 (5.9)		129 (10.7)		401 (33.4)		600 (50.0)		3.3 (0.9)		
	Can telemedicine accurately facilitate the diagnosis of people?	332 (27.6)		478 (39.8)		278 (23.1)		113 (9.4)		2.1 (0.9)		
	Does telemedicine improve the communication between patient and physician?	177 (14.7)		325 (27.1)		420 (35.0)		279 (23.2)		2.7 (0.9)		
	Does telemedicine decrease visits to outpatient clinics?	69 (5.7)		99 (8.2)		454 (37.8)		579 (48.2)		3.3 (0.8)		
	Does telemedicine help increase the speed of performing medical care?	105 (8.7)		203 (16.9)		492 (41.0)		401 (33.4)		2.9 (0.9)		
	Does telemedicine reduce medical errors?	415 (34.6)		474 (39.5)		204 (17.0)		108 (9.0)		2.0 (0.9)		
	Is telemedicine able to provide patients with comprehensive health care?	271 (22.5)		486 (40.5)		302 (25.1)		142 (11.8)		2.3 (0.9)		
	Does telemedicine threaten information privacy?	270 (22.5)		409 (34.1)		331 (27.6)		191 (15.9)		2.4 (1.0)		
	Does telemedicine reduce the costs of providing health care?	87 (7.2)		217 (18.1)		510 (42.5)		387 (32.2)		2.9 (0.9)		
	Are you willing to use telemedicine for your medical diagnosis or follow-up?	189 (15.7)		198 (16.5)		456 (38.0)		358 (29.8)		2.8 (1.0)		
	Telemedicine will improve access to specialized health care for people who live in rural and suburban areas.	153 (12.7)		207 (17.2)		455 (37.9)		386 (32.1)		2.9 (0.9)		
**Perceptions**												
	Do you believe that Jordan has the capacity to adopt and implement telemedicine services?	338 (28.1)		288 (32.3)		306 (25.5)		169 (14.1)		2.3 (1.0)		
	Telemedicine can be integrated within the existing system.	177 (14.7)		319 (26.6)		457 (38.1)		248 (20.6)		2.6 (0.9)		
	Telemedicine is the future of clinical practice.	187 (15.6)		360 (30.0)		427 (35.6)		227 (18.9)		2.6 (0.9)		

### Factors Associated With Knowledge and Attitudes Toward Telemedicine

Univariate analysis demonstrated that having higher educational degrees (*P*<.001), living in urban districts (*P*=.002), and having a higher perception of electronics usage ability (*P*<.001) were associated with higher knowledge of telemedicine. Similarly, having a higher educational level (*P*=.006), residing within the capital (*P*<.001), having a fast internet connection (*P*=.004), and having a higher perception of electronics usage ability (*P*<.001) were associated with more positive attitudes toward telemedicine. Furthermore, female gender was associated with more positive perceptions of the implementation of telemedicine within Jordan (*P*=.007). Table 4 delineates the associations between attitudes, knowledge, perceptions, and different sociodemographic variables.

On another note, it appears that being married (odds ratio [OR] 1.426, 95% CI 1.017 to 1.998; *P*=.04), having children (OR 1.498, 95% CI 1.074 to 2.089; *P*=.02), having comorbidities (OR 1.770, 95% CI 1.244 to 2.517; *P*=.002), and taking medications (OR 1.695, 95% CI 1.212 to 2.371; *P*=.003) were associated with a higher likelihood of telemedicine usage.

**Table 4:** Differences in knowledge, attitude, and perception scores across different sociodemographic variables.

Variables			Knowledge score, mean (SD)		*P* value		Attitudes score, mean (SD)		*P* value		Perception score, mean (SD)		*P* value
**Gender**													
	Male	4.8 (1.5)		.96		30.2 (6.6)		.25		7.3 (2.5)		.007	
	Female	4.8 (1.5)				29.7 (5.9)				7.6 (2.2)			
**Age (years)**													
	18-30	4.8 (1.5)		.41		30.3 (5.7)		.05		7.5 (2.3)		.80	
	31-40	4.7 (1.6)				29.7 (6.5)				7.4 (2.4)			
	41-50	4.9 (1.6)				29.8 (6.5)				7.4 (2.4)			
	51-60	4.8 (1.6)				30.0 (7.1)				7.4 (2.6)			
	61-85	4.6 (1.5)				27.8 (6.5)				7.2 (2.5)			
**Educational level**													
	Primary	4.4 (1.4)		<.001		29.0 (6.6)		.006		7.8 (3.0)		.39	
	High school	4.3 (1.5)				28.9 (7.5)				7.4 (2.7)			
	University	4.8 (1.5)				29.9 (5.8)				7.4 (2.3)			
	Postgraduate (ie, masters)	5.2 (1.4)				31.1 (6.5)				7.7 (2.3)			
**Area**													
	Capital (Amman)	4.8 (1.5)		.23		30.1 (3.1)		<.001		7.5 (2.4)		<.001	
	Central	4.7 (1.3)				29.7 (6.7)				7.4 (2.3)			
	North	4.8 (1.6)				30.6 (6.3)				7.7 (2.2)			
	South	4.4 (2.3)				27.1 (7.4)				6.1 (2.6)			
**Residence**													
	Urban	4.8 (1.5)		.002		30.1 (3.2)		.14		7.5 (2.3)		.05	
	Rural	4.4 (1.7)				29.2 (7.1)				7.1 (2.6)			
**Relationship status**													
	Married	4.8 (1.6)		.74		29.7 (6.7)		.09		7.5 (2.3)		.36	
	Single	4.8 (1.5)				30.3 (5.7)				7.4 (2.5)			
**Number of children**													
	0	4.7 (1.5)		.83		30.1 (6.0)		.64		7.6 (2.3)		.38	
	1	4.8 (1.7)				30.5 (6.9)				7.4 (2.4)			
	2	4.8 (1.6)				29.5 (6.3)				7.2 (2.1)			
	≥3	4.8 (1.6)				29.7 (6.6)				7.4 (2.5)			
**Type of internet connection**													
	4G	4.7 (1.5)		.11		30.2 (6.3)		.004		7.5 (2.3)		.72	
	Fiber	4.9 (1.5)				30.4 (3.2)				7.5 (2.4)			
	Phone packets, others	4.7 (1.7)				28.9 (6.5)				7.4 (2.5)			
**Perceived ability**													
	High (8-10)	4.9 (1.5)		<.001		30.7 (6.0)		<.001		7.6 (2.3)		.11	
	Low (0-7)	4.6 (1.6)				29.0 (6.5)				7.4 (2.4)			

### Predictors of Positive Attitudes Toward Telemedicine

The multivariate linear regression analysis demonstrated that the perceived ability of using electronics was associated with positive attitudes (*β*=0.394, 95% CI 0.224 to 0.563), while living in Southern Jordan predicted poor attitudes toward telemedicine (*β*=–2.896, 95% CI –4.873 to –0.919). Table 5 provides the results of the multivariate regression model. Type, and therefore speed, of internet connection did not influence attitudes levels toward telemedicine.

**Table 5:** Factors predicting favorable attitudes toward telemedicine.

Factors	Linear regression model for attitudes			
	*P* value	B	*β*	Lower and upper 95% CIs for B
Age	.74	–0.007	0.022	–0.051 to 0.036
Gender (female)	.59	–0.199	0.369	–0.922 to 0.525
Number of children	.83	0.026	0.122	–0.214 to 0.266
Ability to deal with computers and tablets	<.001	0.394	0.086	0.224 to 0.563
Presence of comorbidities	.87	–0.110	0.671	–1.427 to 1.207
Taking regular medications	.64	0.294	0.620	–0.922 to 1.510
Being Married	.95	–0.034	0.612	–1.234 to 1.165
High school education	.49	–0.871	1.272	–3.367 to 1.624
University education	.57	–0.699	1.243	–3.137 to 1.740
Postgraduate education	.88	0.188	1.304	–2.370 to 2.746
4G internet	.08	0.859	0.495	–0.111 to 1.829
Fiber internet	.07	0.897	0.491	–0.067 to 1.861
Living in Amman	.08	–0.963	0.546	–2.035 to 0.109
Living in Central Jordan	.09	–1.064	0.629	–2.298 to 0.170
Living in Southern Jordan	.004	–2.896	1.008	–4.873 to –0.919

## Discussion

### Principal Findings

In summary, we demonstrated that about one-half of our studied population were aware of the existence of telemedicine, while only a small minority declared having observed or ever used the technology. Furthermore, more than one-half of the population were willing to use telemedicine. Overall, attitudes toward telemedicine were positive. However, participants questioned its capacity to reduce errors, make accurate diagnoses, or provide comprehensive care. Moreover, telemedicine was perceived as a threat to privacy and impractical within Jordan’s current resource capacity. Attitudes and degree of knowledge were typically higher in those with higher education levels, who perceived themselves as competent electronics users, and who lived in metropolitan regions. Regression analysis showed that perceived competency with electronics was a positive predictor of favorable attitudes, while living in rural areas (Southern Jordan) was a negative predictor. Also, a higher likelihood of using telemedicine was associated with having children, being married, having previous comorbidities, or taking medications.

To the best of our knowledge, this study is the first of its kind in Jordan. We aimed to explore the levels of awareness, knowledge, and concerns of the general public toward telemedicine in an attempt to closely inspect the understanding of its potential recipients and providers of the concept of the technology and further evaluate the possibility of incorporating it into the current health system. Within the regional literature, studies conducted in Saudi Arabia and Egyptian populations showcased similar results to those of our study [30,31]. Similar results were also found among citizens of the United Arab Emirates [17]. The aforementioned populations had positive perceptions of telemedicine, its benefits, and its applications within the health care system. However, significant proportions of both populations agreed, to an extent, that telemedicine poses a threat to patients’ privacy and may increase medical errors. Throughout the literature, especially studies conducted in response to the pandemic, it appears that the utility of telemedicine during disasters, its ability to reduce transportation time and costs, and its capacity to improve patient-doctor communication are consistently appreciated [30]. On the other hand, older Asian adults (ie, Chinese, Malaysian, and Indian participants) reported negative perceptions toward the utility and usefulness of telemedicine [34]. Nonetheless, despite the general positive perception of telemedicine, the literature describes that the most commonly reported individual hurdles to successful implementation of telemedicine technology worldwide were related to lack of awareness [35-37] and technical literacy [38,39]. Tackling those factors early through promotion of telemedicine services and enhancing electronic use would likely facilitate smooth integration of the technology into the current system.

In terms of attitudes, our participants demonstrated positive perceptions toward telemedicine. They believed that it has the capacity to decrease transportation costs and the number of medical staff required. They also believed that telemedicine can smoothen health care logistics and facilitate better health care for older adults. Mutual economic benefits for patients and health care providers were evidently recognized in successful telemedicine projects in Jordan [21]. Furthermore, an integrative review [40] displayed evidence supporting the facilitation of delivered care and improved health outcomes for older adults through the media of telemedicine. Such a value is particularly essential in the era of COVID-19 as well as among older adults, who possess a higher risk of infection and complications from the disease and could benefit from reducing hospital visits whenever possible [41]. This is further relevant to Jordan’s medical landscape, as it is characterized by patient congestion and long waiting times at nearly every public medical institution. These factors could increase the risk of infection for older adults, thus positively affecting the perceptions of the caretakers and relatives of older adults toward the usefulness of telemedicine.

Moreover, Jordanian participants believed that telemedicine helps in reducing outpatient visits, increasing the speed of delivering health care services, and providing more comprehensive care to underserved regions. Also, they perceive it as a useful tool during a pandemic similar to that of COVID-19. Similar statements were also observed from residents of Saudi Arabia and Egypt [30,31]. In conjunction with social distancing and work-from-home policies, telemedicine represented a viable and sustainable option to migrate health care from hospitals to homes, which promoted reductions in outpatient clinic visits and further aided in the endeavors to mitigate COVID-19 transmission [6]. In addition, resource-limited countries often experience a scarcity in health care providers, particularly specialists. Therefore, telemedicine could also provide a novel approach to improve access to more specialized wellness programs for vast majorities of people as well as revitalizing weakened public health services while significantly reducing costs [42].

On the other hand, our study participants expressed a multitude of concerns toward telemedicine. These concerns were mostly pertaining to accuracy of diagnosis, medical errors, and information privacy. Diagnostic accuracy through telemedicine services has been thoroughly evaluated in the literature. Most studies reported high accuracy in specialties where telemedicine is viable, although variations across literature are still evident. Most notably, those specialties were dermatology [43] and ophthalmology [44,45].

Although some studies associate the rapid expansion of telemedicine technology in COVID-19 with an increased incidence of medical errors [46], a root cause analysis of medical errors done by the Agency for Healthcare Research and Quality Patient Safety Network [47] revealed that most incidents were related to human factors such as communication failures between health care personnel. Telemedicine, when implemented properly and utilized by competent personnel, could serve as a tool to help mitigate those errors by providing continuous surveillance to delivered care, especially in critical settings such as that of the intensive care unit [48].

Maintaining privacy and information security was a challenging conundrum that was threatened and prominently highlighted through the era of rapid expansion of telemedicine services during COVID-19. Such challenges were manifested as a lack of controls or limits on the collection, use, and disclosure of sensitive personal information, repeated cyberattacks, and rapid spread of ransomware and went as far as suspected death in patients [49]. Such hazards should be dealt with by a multidisciplinary approach including health care organizers, policy makers, and information technology specialists and by the implication of guidelines and rules that regulate the use of telemedicine services similar to those of the Health Insurance Portability and Accountability Act regulating traditional medicine. Efforts should be concentrated at training both the health care provider and the patient about the necessary precautions to prevent any breach of confidentiality and at enforcing end-to-end data encryption of patients’ information and records in order to mitigate those adverse effects and sustain a culture of security [50].

In our study, higher rates of telemedicine use were significantly higher among patients with comorbidities and those taking chronic medications. Such results could be largely explained by the fact that, in 2020, due to COVID-19 restrictions, the Ministry of Health, along with the Royal Medical Services and university hospitals, offered primarily medication refill services through telemedicine programs [51]. This largely promoted ease of accessibility and was a good substitute to in-person appointments.

Among the studied cohort, participants who were married and had children had a higher likelihood of telemedicine usage. The particular use of telemedicine services within the context of a marriage appears to be a solution for a myriad of obstacles that otherwise would prevent couples from seeking therapy such as childcare, scheduling difficulties, and stigma. Providing couples treatment via telemedicine services was reported to address these obstacles and improve accessibility to health care services [52,53]. Additionally, the use of telemedicine services to perform regular prenatal counseling was associated with high rates of feasibility and satisfaction [54]; such factors could encourage multiparous women who might be more lenient when it comes to prenatal visits to have a positive perspective regarding telemedicine within this context, to save time and effort.

More than one-half of the studied population believed that Jordan cannot successfully integrate telemedicine into the current health care system. This may be due to resource scarcity, as it remains a major hindrance for the implementation of telemedicine in Jordan. Considering its position as a country with limited resources and a high poverty rate of 24.1% [55], the implementation of telemedicine in an ideal manner in which high satisfaction and wide coverage of various medical specialties for the majority of the population are achieved remains a struggle that necessitates urgent policy changes, making the development of the health care sector as a whole of upmost priority in the country in order to facilitate the path for optimal effectuation of telemedicine.

Another reason for such negative perceptions of telemedicine is the misleading status of the technological infrastructure in Jordan. Although around 67% of the Jordanian population utilize the internet [56], internet quality and speed might be the greatest hindrance to proper implementation of telemedicine, particularly in remote areas. The cheapest home internet subscription in Jordan costs around US $50, and, considering that the average minimum wage is around US $370, people from a lower socioeconomic status will be discouraged from having the necessary resources to benefit from telemedicine [57]. It should be noted that only 33% of Jordanians owned a personal computer or laptop as of 2017 [58]. Interestingly, our prediction model did not correlate the type of internet with attitudes toward telemedicine. This may be a result of the survey distribution process, as it was primarily online, thus targeting participants with better internet connections.

### Limitations

Our results should be considered with caution due to the following limitations: the cross-sectional study design and its implications, close-ended nature of the questionnaire that may miss certain participants’ responses, and sampling technique that may have missed certain groups of the Jordanian populace (eg, older adults and technologically illiterate individuals).

### Conclusions

In light of these findings, Jordanians have favorable and positive perceptions of telemedicine that are similar to other populations within the same region. Nonetheless, concerns with regards to privacy, medical errors, and capacity for making accurate diagnoses are prevalent. Despite the generally positive perceptions toward and willingness to use telemedicine, Jordanians believed that telemedicine cannot be easily integrated within the current system nor do they have resources to adopt it.

## Abbreviations

OR: odds ratio
